# QSAR DataBank repository: open and linked qualitative and quantitative structure–activity relationship models

**DOI:** 10.1186/s13321-015-0082-6

**Published:** 2015-06-25

**Authors:** V Ruusmann, S Sild, U Maran

**Affiliations:** Institute of Chemistry, University of Tartu, Ravila 14a, 50411 Tartu, Estonia

**Keywords:** QSAR, QSPR, QSTR, QsarDB, QDB, Digital repository, Digital object identifier, ODOSOS, Predictive model, DSpace

## Abstract

**Background:**

Structure–activity relationship models have been used to gain insight into chemical and physical processes in biomedicine, toxicology, biotechnology, etc. for almost a century. They have been recognized as valuable tools in decision support workflows for qualitative and quantitative predictions. The main obstacle preventing broader adoption of quantitative structure–activity relationships [(Q)SARs] is that published models are still relatively difficult to discover, retrieve and redeploy in a modern computer-oriented environment. This publication describes a digital repository that makes in silico (Q)SAR-type descriptive and predictive models archivable, citable and usable in a novel way for most common research and applied science purposes.

**Description:**

The QSAR DataBank (QsarDB) repository aims to make the processes and outcomes of in silico modelling work transparent, reproducible and accessible. Briefly, the models are represented in the QsarDB data format and stored in a content-aware repository (a.k.a. smart repository). Content awareness has two dimensions. First, models are organized into collections and then into collection hierarchies based on their metadata. Second, the repository is not only an environment for browsing and downloading models (the QDB archive) but also offers integrated services, such as model analysis and visualization and prediction making.

**Conclusions:**

The QsarDB repository unlocks the potential of descriptive and predictive in silico (Q)SAR-type models by allowing new and different types of collaboration between model developers and model users. The key enabling factor is the representation of (Q)SAR models in the QsarDB data format, which makes it easy to preserve and share all relevant data, information and knowledge. Model developers can become more productive by effectively reusing prior art. Model users can make more confident decisions by relying on supporting information that is larger and more diverse than before. Furthermore, the smart repository automates most of the mundane work (e.g., collecting, systematizing, and reporting data), thereby reducing the time to decision.

**Graphical abstract:**

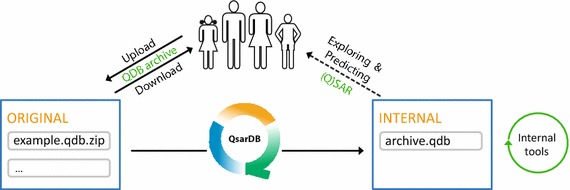

## Background

Chemo-informatics has been developed to address chemical and experimental data. Different bits of data have been put into context, i.e., annotated, establishing valuable sources of information in the form of chemical and biochemical databases. This information is used for systematic analysis using various statistical and machine-learning approaches. The result of such abstractions is usually provided in the form of in silico models. These models in chemistry and related areas are known by the common name quantitative structure–activity relationships, (Q)SARs, and have a wide range of complexities and functions. SAR models seek to express relationships between chemical structure and physical or chemical processes that occur in organisms or in the environment. QSAR models extend this approach to express relationships in mathematical terms so that it is possible to compute qualitative and quantitative values. (Q)SARs have been at the forefront of explaining obscure physical, chemical, and biological phenomena for almost a century. Studies of (Q)SAR models during the past two or three decades have not challenged the underlying assumptions very much but have contributed significantly to the mathematical methods and tools needed for more complex analysis. Despite being a valuable tool for explaining physicochemical and chemico-biological mechanisms in nature and living organisms, the real usefulness of (Q)SAR is as an application tool for decision support [1]. However, this transition has been inhibited by conflicting interests between major stakeholders. Researchers and reviewers, who are collectively in charge of model development, care more about the methodology and reproducibility issues than the ease of use. At the same time, end users, who are in charge of model consumption, care more about the applicability and reusability issues than the overall theoretical foundation. This dichotomy has prevented meaningful, concerted action to organize published (Q)SAR models in an open and systematic way. This work describes a digital repository platform called the QsarDB repository [2], which collects in silico (Q)SAR-type descriptive and predictive models that are represented in the QSAR DataBank data format (QsarDB data format) [3]. This approach makes the knowledge easily accessible, citable, locatable and usable for most common research and applied science purposes.

The QsarDB repository is a domain-specific digital repository for archiving published (Q)SAR models in the QsarDB data format and assigning globally unique uniform resource identifiers (URI) to them. To the best of the authors’ knowledge, this problem is still unsolved in the public domain. The QsarDB repository is designed as a public service aimed at communicating and publishing (Q)SAR models for the widest audience possible, i.e., for both model developers and model users. The upload and download of QDB archives is open for all. However, there is a quality-control process over submissions to ensure conformance with quality criteria [3] that are dictated by the hosting entity. Additionally, all QDB archives must be associated with a “source”, such as a publication or another resource identifier. The publication, which is easy to follow via hyperlink, is regarded as the primary documentation of the QDB archive and provides both technical information (e.g., the description of the modelling process) and the user information (e.g., the characterization of the intended applicability domain and suggested mechanistic interpretation).

The QsarDB repository falls into the category of content-aware or “smart” repositories because it adds value by making the content of QDB archives explorable and useable online. It is designed for interactive use, so that end users can execute desired operations directly on deposited models. The solution is based on extending the capabilities of one of the most popular digital repository platforms called DSpace [4]. This guarantees that the QsarDB repository supports all of the state-of-the-art digital repository requirements, data-exchange interfaces and standards.

(Q)SAR models are made visible and available in two ways. First, the repository approach is designed for the long-term preservation of (Q)SAR models in a specific data format. The underlying assumption is that every published model has been previously screened by experts in the field (e.g., published in a peer-reviewed journal) and is considered to be universally useful over long periods of time. Second, the integrated modelling environment approach is designed to make (Q)SAR models available that have been developed on a specific web-based or standalone platform. In this case, the published models may or may not have been reviewed by third parties.

In the first category the only public repository of in silico QSAR-type models that bears similarity to the QsarDB repository is the EC JRC-IHCP QSAR Model Reporting Format Inventory [5], which accepts models in (Q)SAR Model Reporting Format (QMRF) [6]. At the time of writing, this repository contains 80 models documented according to specific rules. However, the QMRF data format [6] is functionally very different from the QsarDB data format [3]. As the name suggests, QMRF documents are text-based reports about the key aspects of a model. It takes considerable effort to convert them into a structured, machine-readable form. The QsarDB repository contains an example collection of QDB archives [7] that have been adopted from the QMRF inventory and also have scientific publications describing the model(s).

The choice of web-based integrated modelling environments is much broader. The list of model databases that also provide model development and/or prediction functionality includes OCHEM [8], Chembench [9], AMBIT2/OpenTox API [10] and GUSAR from the NIH [11]. Additionally, notable collections of (Q)SARs are implemented and distributed as integral parts of stand-alone software solutions with free or limited access. This type of collection includes EPI Suite™ from the US EPA [12, 13], X3logP [14], VEGA [15], ChemProp [16], ToxTree [17–19], QSARINS-Chem [20], the OECD QSAR Toolbox [21], etc. The limitation of these software solutions is related to the access to the original data sets used for the model development and also to the limited extension capabilities.

## Construction and content

### Repository platform

The DSpace software [4] is a functionally complete digital repository platform that conforms to most common interoperability standards, such as the Open Archives Initiative [22]. Over the years, it has attracted a large community of developers and users ranging from academia to multinational corporations [23]. DSpace in its default configuration supports only generic content, such as texts documents, multimedia documents (music, photos, and videos), etc. However, it meets the criteria of a semi-smart repository because all supported documents are parsed, harvested for metadata and/or indexed for full-text search purposes. DSpace can be made “smarter” about new document data formats by extending its codebase with custom Java application code. The QsarDB extension has two major goals. First, the content of submitted QDB archives must be properly supported and integrated into the DSpace metadata framework. Second, if possible, the user interface should be enhanced with interactive QsarDB-specific widgets. The integration with the QsarDB data format [3] is based on the QsarDB Java reference implementation (JRI) [24] and middleware libraries [25].

**Figure 1:**
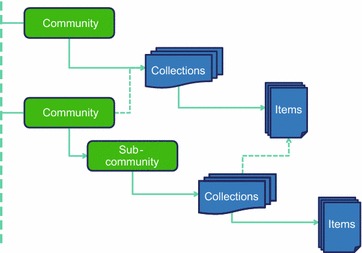
Organization of the repository content into logical levels.

The DSpace data model organizes repository content at two logical levels [26]. The higher-level unit of organization is called Community, and the lower one is called Collection (Figure 1). As the name suggests, Community addresses actors such as persons and/or institutions, whereas Collection addresses actual content. This system is easily adaptable to various organizational and community structures. The QsarDB repository currently employs a simple two-level hierarchy, in which Community represents a research group (or a project) and Collection represents related content.

In DSpace terminology, archived objects are called Items. A single Item consists of a metadata record and one or more documents. The QsarDB repository requires every Item to contain exactly one QDB archive, which may be accompanied by appropriate reference materials (e.g., author copy of the original publication, QMRF document(s), other normative and regulative documents, etc.). Every Item is owned by one parent Collection, but it is possible for an Item to be shared between multiple Collections. Such a one-to-many relationship gives enough flexibility to organize the repository content in a flexible and intuitive manner. A typical example is related to the collaborative nature of (Q)SAR research, in which a single publication is typically authored by multiple research groups.

### Persistent digital identifiers

DSpace automatically assigns [26] unique and persistent identifiers to each end user-oriented resource (i.e., Community, Collection, or Item). HDL identifiers are provided by the Handle System [27], which is a service for performing translation between persistent identifiers and actual up-to-date URIs. The QsarDB repository has been assigning HDL identifiers via CNRI services [28] since May 1, 2012.

The most widely known Handle System implementation is the Digital Object Identifier (DOI) system, which is managed by the International DOI Foundation [29]. DSpace has implemented DOI support for Items starting from version 4.0. The QsarDB repository assigns DOIs to Items starting from August 21, 2014. The reservation of DOIs is performed via DataCite [30, 31] services. Complete reliance on persistent identifiers helps create and maintain long-lasting promises about the discoverability and usability of the QsarDB repository content.

### Model sources

**Figure 2:**
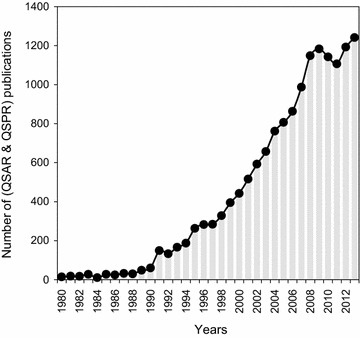
Number of QSAR and QSPR publications in the peer-reviewed literature, years 1980–2013.

The QsarDB repository is designed for models produced with all statistical and mathematical algorithms that qualitatively or quantitatively express the relationship between the chemical structure and the responses of a compound. This information includes chemico-biological activity (QSAR), physicochemical properties (QSPR), toxicity (QSTR), metabolism (QSMR), reactivity (QSRR), retention (QSRR), permeability (QSPR), pharmacokinetics (QSPR), bioavailability (QSBR), binding (QSBR), etc. The primary sources of the data and models are publications in scientific journals. According to the Web of Science™ database, there are thousands of publications in the peer-reviewed literature per year that refer to the terms “QSAR” or “QSPR” (Figure 2). It is rather difficult to estimate the actual number of (Q)SAR models from this result because the query may be incomplete and there are publications that simply refer to existing models or propose several new models at once. The development of models is continuously fuelled by novel experimental datasets and research in statistical and machine-learning methods.

However, scientific journals are not the only source of models for the QsarDB repository. In principle, a source can be any medium that provides a model object and adequate documentation about the hypothesis, model development and validation process, the description of the results (e.g., suggested mechanistic interpretation, applicability domain, etc.). The Open Notebook Science challenge on predictive solubility [32] is a good example of using Wiki-based scientific notebooks as a source of (Q)SAR models (see the “Current status and use examples of the QsarDB repository” section).

### Repository implementation

DSpace represents deposited data as Items. An Item consists of metadata records and one or more documents (i.e., files) [26]. The Item is organized into named Bundles of byte streams. The purpose of each Bundle is stipulated according to DSpace conventions. Each Item has the “ORIGINAL” Bundle that contains the verbatim copy of the user-deposited documents. The repository software may use the “ORIGINAL” Bundle to derive secondary Bundles. For example, the DSpace software extracts the plain-text data from deposited documents to a “TEXT” Bundle that becomes the basis for full-text searching.

**Figure 3:**
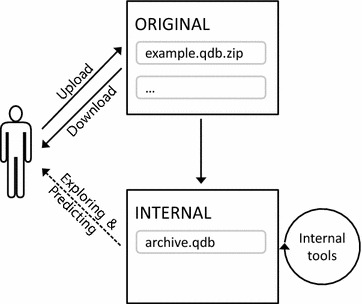
Lifecycle of the QDB archive. A file uploaded by a submitter is located in the public data repository and named the “ORIGINAL”. The QsarDB repository creates a working version of this file during the submission workflow, which is placed in the internal data repository “INTERNAL”. The working version can be developed further using the built-in QDB repository tools.

This design (in which original and derived data are kept separate) is very useful for QsarDB repository needs. First, the original QDB archive (and any number of accompanying documents) is deposited in the “ORIGINAL” Bundle. Then, a copy of the original QDB archive is processed and optimized to produce a variant that is more suitable for internal processing needs. This derived QDB archive is stored in the “INTERNAL” Bundle (Figure 3). The internal representation can be incrementally modified as the system evolves. Typical modifications are the computation of statistical or applicability-domain parameters according to harmonized protocols.

DSpace associates end user-oriented resources with metadata records to make them suitable for easy browsing and searching [26]. The default metadata schema is the Dublin Core metadata standard [33]. However, the proper description of Items that feature complex content requires the combination of multiple metadata schemas. DSpace is open for extension with additional metadata schemas. For example, DSpace has a plugin system to translate data between the internal metadata format and external data and metadata formats. The QsarDB repository uses it for QSAR-specific metadata.

### Metadata and provenance information

**Figure 4:**
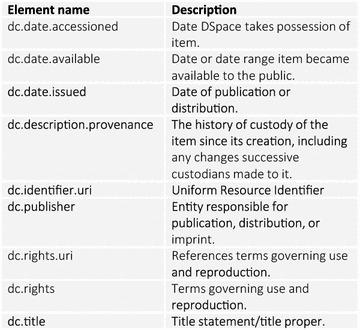
Dublin Core metadata elements and their descriptions used in QsarDB.

The QsarDB data format features incremental design [3], where the introduction of every one or two Container types adds a new layer of complexity. Therefore, it is practical to characterize QDB archives using a set of metadata schemas that are “activated” one by one. DSpace relies heavily on a subset of Dublin Core metadata schema. Every Item is required to have a Dublin Core record [26] with most critical metadata (Figure 4). For example, the value of the “dc.title” element corresponds to the bibliography reference of the original publication. It is automatically generated based on the bibliography metadata schema during the Item submission (see the “Submission workflow” section). The value generation uses the American Chemical Society reference style [34] for the sake of uniformity.

**Figure 5:**
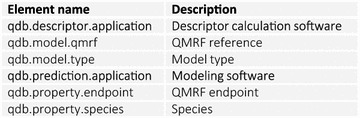
QsarDB-specific metadata elements and their descriptions.

**Figure 6:**
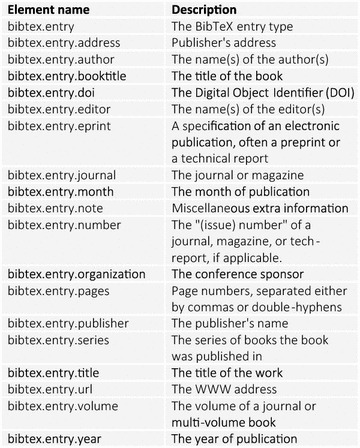
BibTeX-specific metadata elements and their descriptions.

The QsarDB repository introduces two additional metadata schemas. First, the QsarDB metadata schema reflects the contents of the QDB archives, such as the endpoint, the type of models (e.g., regression or classification), the name and version of the software that was used for the descriptor calculations and statistical analyses (Figure 5). Second, the bibliography metadata schema (Figure 6) reflects all of the information that is known about the source of the (Q)SAR model. The bibliography metadata schema is designed after the BibTeX data format [35]. The web interface is equipped with widgets that provide browse and search functionality, depending on the selected metadata element.

### Licensing of QDB archives

The authoring of QDB archives requires conscious effort, thereby making it creative work. The author of the QsarDB archive must decide whether to make work publicly available and if so, under what terms. DSpace makes the distinction between deposit and distribution licenses clear. The deposit license is granted to the hosting organization and specifies the rights of the host. The distribution license specifies the rights of the end users who download the archive content.

The default deposit license in DSpace is a non-exclusive license that confirms that the depositor owns the copyright to the submitted content and that grants the hosting organization permission to make the submitted content publicly available and to take necessary steps for preserving it. After submission, the author of the QDB archive will retain all rights for the submitted content. The archives deposited to the QsarDB repository are open access by default. Authors are hereby encouraged to license their work under the Public Domain (or Creative Commons) license so that the rights for using the content are explicitly defined to the end users.

### Submission workflow

**Figure 7:**
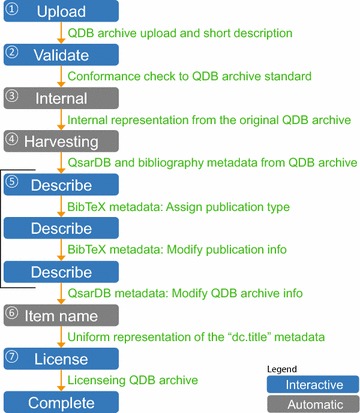
Item submission workflow.

All submissions to the QsarDB repository undergo a special purpose workflow, which performs upload, validation, optimization, metadata-extraction, metadata-description, standardization, and license-selection steps (Figure 7):

1. The upload step uses the standard DSpace form to transfer the QDB archive and optional supplementary information (see the “Repository platform” section). The supporting information is not validated in any way.
2. The validation step performs functional validation of the QDB archive content. The submitter must specify the target compliance level: “basic”, “intermediate” or “advanced” (see details in the “Quality Control of QDB archive file” section). If the validation fails, the submitter is prevented from continuing with the submission workflow.
3. The enhancement and optimization step generates the internal representation from the original QDB archive, as described in the “Repository implementation” section. This operation is performed automatically by the QsarDB repository.
4. The metadata-extraction step initializes basic QsarDB and bibliography metadata records with element values that can be readily harvested from the QDB archive content. Again, this operation is performed automatically by the QsarDB repository to reduce the need for manual data entry in the next step.
5. The metadata-description step presents web forms with prefilled QsarDB and bibliography metadata. The submitter can review and modify existing elements and/or add missing elements. The description step also ensures that all mandatory metadata elements are described before the user can proceed to the next step.
6. The Item-naming step is used for the uniform representation of the “dc.title” metadata element. Its initial value is automatically generated (see the “Metadata and provenance information” section) based on the bibliography metadata that was provided in the previous step.
7. The licensing step provides an interactive form to the submitter to assign a license to the Item. To ensure the maximal freedom to reuse the data, the use of the Public Domain license is encouraged.

The review policy of the target Collection determines what occurs upon completion of the submission workflow. The Item may either become immediately visible to the general public or optionally enter an additional review process. The review is typically conducted by the “maintainer” of the Community of the target Collection. This person can view and edit metadata records and make a final decision about whether the Item should be accepted or rejected. In addition, the access to the Item can be set to restricted or embargoed.

### Quality control of the QDB archive file

The second step of the submission workflow performs automated quality control of the QDB archives [3]. The submitter can choose between three validation levels: “basic”, “intermediate” or “advanced”. Validation can (1) pass cleanly, (2) pass with one or more warnings or (3) fail with at least one error. Warnings represent minor deficiencies (e.g., an optional Container attribute is missing) or general suggestions about how to improve the quality of the work. Errors represent serious deficiencies (e.g., a required Container attribute is missing) or inconsistencies (e.g., broken Container relationships).

Basic validation (“sanity testing”) ensures that the QDB archive meets all structural and well-formedness requirements. The checks attempt to cover as many syntactic and semantic features as possible. Generic checks ensure that the Containers have their required attributes defined, that all declared Cargos are present in the archive, and that all system Cargos can be parsed without errors. In addition to generic checks, there are Container type-specific checks. For example, the validity of the CAS number and InChI code is checked for Compounds, the presence of mathematical representation is checked for Models, and so on. Another important type of check is the Container relationship check. For example, a Model container must refer to an existing Property via a strong relationship (see [3]). Similarly, Property, Descriptor and Prediction containers must have “values” Cargo, and each value in the Cargo must refer to an existing Compound via a weak relationship (see [3]).

Intermediate validation (“reusability testing”) ensures that the Compound, Property and Descriptor containers can be uniquely identified. This validation level requires that all Compounds must have the InChI attribute, that the Properties must have both endpoint attributes with their units given as UCUM Cargo, and that the Descriptors must have the application attribute.

Advanced validation (“reproducibility testing”) ensures that the mathematical representation of the model can be evaluated and that the model is reproducible using the raw data in the archive. At this validation level, each model is loaded, and its Predictions are re-evaluated with the included Descriptor data. The calculated Prediction values are compared with the Prediction values included in the archive.

## Utility and discussion

### QDB archive view

The content of the repository can be conveniently accessed via the DSpace web interface. Due to the complexity of (Q)SAR models and the related data, additional functions have been developed that improve the utility of the deposited QDB archives for the scientific community. Basic repository features include browsing and searching of the repository content using available metadata fields, such as model authors, model titles, endpoints, species, etc. Browse and search can be performed over all of the repository content or can be limited to a given Community or Collection.

**Figure 8:**
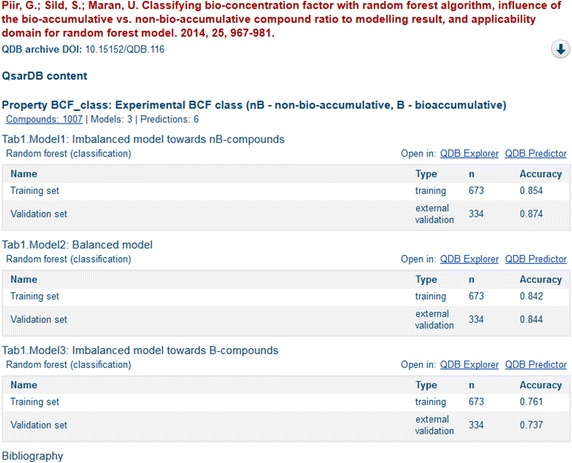
Fragment from Item view (XMLUI interface) that shows an example of the Models information contained in the QDB archive.

Detailed information about a specific QDB archive is displayed on its Item view page. The standard view includes a tabular overview about the Item’s metadata, as well as download links to its content (Figure 8). The additional features provide high-level overviews of the deposited QDB archive, which summarize the Properties included in the archive. Each Property summary includes the list of related Models, along with their Prediction statistics and relevant bibliography references. The Item view also adds links for each model that take the user to web applications that allow for detailed analysis and visualization of the Model (QDB Explorer) and online predictions (QDB Predictor).

### QDB explorer application

The QDB Explorer web application presents available information that is related to the (Q)SAR model. This application retrieves model data using internal web services and renders this information in the web browser. In the QDB Explorer view, the dataset is presented as a data table, which includes essential compound attributes (e.g., Id, Name), experimental and predicted property values and descriptor values. Primarily, the QDB Explorer shows raw data from the archive, but certain secondary data are also calculated by the repository, including prediction errors, leverages and Mahalanobis distances for applicability-domain analysis. The data table is interactive and allows for reordering table rows by column and filtering table rows by the membership to training, validation and testing sets. In addition, tooltips at the column headers with additional metadata for columns, as well as tooltips with structural information for every compound, are available.

The QDB Explorer also includes visualization tools for inspecting data distributions and interdependencies. The visualization of property data depends on the model type. For example, classification models are characterized by confusion matrices, whereas regression models are characterized by scatterplots with experimental versus predicted values and histograms for the property values, etc. The prediction errors are depicted using residual plots (i.e., scatterplots with experimental property versus residual error). Each descriptor involved has a scatterplot with the descriptor versus the experimental property, as well as a histogram of the descriptor values. The applicability-domain analysis is possible with Williams and Gramatica plots (also known as an Insubria graph [36]), in which the distances between compounds can be represented as leverage or Mahalanobis distances. All of the scatterplots mentioned above are also interactive, and hovering above a data point will show a tooltip with structural information about the compound of interest.

### QDB predictor application

The QDB Predictor application enables the use of models for predictions and for understanding how independent variables (i.e., descriptors) contribute to the calculated property value. The QDB Predictor has various options for entering input data to perform the prediction. The input data can be a SMILES string or InChI code for models that use Chemistry Development Kit (CDK) [37] descriptors (which can be extended to other descriptor calculators in the future). In this case, the repository calculates descriptors and evaluates the model. If the descriptor calculation is not possible, then the descriptor values can be entered manually via the descriptor-input widget. It is also possible to browse compounds in the data set and to repeat predictions using the raw data contained in the archive. Predictions are accompanied by similarity analysis, in which the relevant data about the most similar compounds from the dataset are presented for reference. The similarity criterion is the Euclidean distance calculated using the descriptor values of the compounds in the present setup. The visual comparison of the query compound with similar compounds and their experimental and calculated property values provide useful insights into the reliability of the prediction.

### Web services

**Figure 9:**
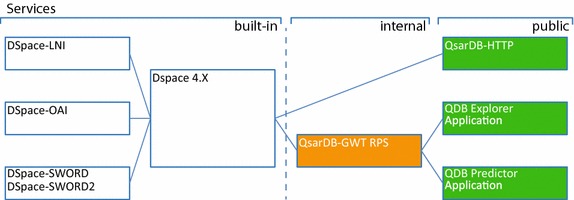
QsarDB repository by the end user-oriented services. On the *left* are the built-in services (*white background*) that operate on the Item level. On the *right* are the internal (*orange background*) and public services (*green background*) that run at the embedded Item level of the QDB archive.

The QsarDB repository provides two types of web services that implement (Q)SAR-specific functionality based on deposited QDB archives (Figure 9). One group of services represents public application of programming interface (API). These services can be consumed by web applications and/or standalone applications. The second group of services is strictly for internal use. These services are consumed by widgets that are integrated into the web interface. With the help of such widgets, it is possible for end users to visualize and analyze models very conveniently, directly from their web browser.

The public web service API is documented on the QsarDB website [38]. It can be used for predictions with a subset of deposited (Q)SAR models. The prediction service is simple to use and requires only a chemical structure for the query compound to produce the predicted value. The prediction service addresses the computational details, which include (1) preparing a suitable chemical structure representation for the calculation of the descriptors, (2) calculating the descriptor values, and (3) evaluating the model using the calculated descriptor values. Currently, this functionality is supported only for limited sets of models that use CDK descriptors [37].

### Current status and use examples of QsarDB repository

At the time of writing, the QsarDB repository included 128 QDB archives: 98 have models, and 30 only have experimental data. These 98 QDB archives include 304 unique models. The population of the QsarDB repository has followed an historical development path within the group for organizing experimental data and (Q)SARs, and it has not been systematic. Therefore, the QDB archives and models are biased towards toxicity endpoints, but this is expected to change in the future.

The authors’ work on data curation [39] gives an example of the Collection hosting the primary experimental data and (Q)SAR models for *Tetrahymena pyriformis* toxic-potency data as the 50% population growth-impairment concentrations (IGC_50_) [40]. The proposed timeline approach for data curation operates on several (i.e., two or more) original data sets represented in the form of QDB archives, and it produces a consensus data set accompanied by an extensive data-curation effort [39]. Having the primary data organized in QDB archives allows for effective and semi-automated data curation. From those 85 archives, all of them contain experimental data, and 55 also have (Q)SAR models.

As already discussed in the “Model sources” section, the (Q)SARs must have a scientific publication as a major model uploading policy because scientific publication includes the full “metadata” about model development and the valuable background information required to understand the subject area under study. However, the important fact is that the model and its related information are well documented, which can also be achieved by other means. Consequently, a second example of use comes from the Wiki-based scientific notebooks from the Open Notebook Science team lead by Jean-Claude Bradley (†) of Drexel University (Philadelphia, PA) and Andrew S.I.D. Lang of Oral Roberts University (Tulsa, OK). Open Notebook Science established a Community at the QsarDB repository [41], including a Collection of original models for predicting the Abraham model solvent coefficients and descriptors and the ONS Melting Point Model 010 [42]. These models were all from Useful Chemistry and Open Notebook Science Challenge projects [43]. All of these models have been developed using open tools that allow use of the full capabilities of the QDB Predictor, and the prediction of values from these structures and models has been performed since their upload in May–June 2012.

## Conclusions

The QsarDB repository is a practical resource and tool for the QSAR community. It is designed around the idea that (Q)SAR data, information and knowledge should be easy to communicate and collaborate on. The QsarDB repository is tailored to a variety of user roles and experience. For model developers, it provides a simple means to register, upload and communicate models, making the models uniquely citable and the supplementary material of scientific publications interactive. For model end users (and also for casual visitors), it provides simple web interfaces and services to quickly and easily locate models of interest, along with the related data. Raw data can be quickly summarized and visualized using the data dashboard view. Models can be visualized, analysed numerically or used for prediction in single-prediction or batch-prediction mode. Predictions can be formatted to make them suitable for decision support. Advanced end users can download and reuse models at local locations for more advanced study and evaluation.

### Availability and requirements

QsarDB repository content is freely accessible at the following URL: http://qsardb.org/. The source code for the QsarDB libraries and the modified version of the DSpace software are available via GitHub: http://github.org/qsardb
